# Colonic Malignant Melanoma: ^18^F-FDG PET/CT Findings

**DOI:** 10.4274/mirt.65807

**Published:** 2018-10-09

**Authors:** Eser Kaya, Tamer Aksoy, Ahmet Levent Güner, Hakan Temiz, Erkan Vardareli

**Affiliations:** 1 Acıbadem Mehmet Ali Aydınlar University Faculty of Medicine, Department of Nuclear Medicine, İstanbul, Turkey

**Keywords:** Colon, malignant melanoma, 18F-FDG PET/CT

## Abstract

Primary malignant melanoma occurs most often in the skin and much less frequently in the choroid layer of the eyes, in the leptomeninges, oral cavity, nasal mucosa, pharynx, esophagus, bronchus, under the nail and vaginal or anorectal mucosa. Primary melanoma of the gastrointestinal tract has been confirmed for lesions occurring in the esophagus, stomach, small bowel, and anorectum through several published reports, as these are the areas where melanocytes normally exist. The occurrence of primary malignant melanoma in the colon is relatively rare, because melanocytes are embryologically absent in the large bowel. Herein we report a patient whose colonic malignant melanoma was diagnosed and disseminated metastatic lesions were revealed with ^18^F-FDG PET/CT scan. There were multiple nodular lesions showing increased ^18^F-FDG uptake in both lungs. There was a soft tissue lesion with slightly increased ^18^F-FDG uptake, which extended to the intraluminal region of the thoracic esophagus. Increased metabolic activity was detected in the asymmetric stomach wall thickening site and in a soft tissue lesion located on the gall bladder wall that was filling the lumen. Multiple hypodense/hyper-metabolic lesions were identified in the liver. Multiple hyper-metabolic polypoid soft tissue lesions were visualized in almost the entire colonic segments. Multiple hyper-metabolic peritoneal implants were noted in all abdominal quadrants. Increased ^18^F-FDG uptake was detected at the right surrenal gland soft tissue lesion. There was a hyper-metabolic soft tissue lesion on the posterior wall of the rectum. Hyper-metabolic lytic lesions were seen at the thoracal and lumbar vertebrae, left scapula, left iliac bone, sacrum and left femur. There was no evidence of ^18^F-FDG avid skin lesions in both attenuation corrected and non-corrected images.

## Abstract (tr)

Primer malign melanom sıklıkla deride ortaya çıkmaktadır, daha az sıklıkla da, gözün koroid tabakasında, leptomeninkslerde, oral kavitede, nazal mukozada, farinkste, özefagusta, bronşlarda, tırnak altında, vagende ve anorektal mukozada izlenmektedir. Primer gastrointestinal sistem melanomu, melanositlerin normal olarak bulunduğu yerlerden özofagus, mide, ince barsakta ve anorektumda bildirilmiştir. Melanositler embriyolojik olarak kalın barsakta bulunmadığından, kolonda primer malign melanom oluşumu nadirdir. Bu makalede kolon malign melanoma tanısı alan ve ^18^F-FDG PET/BT taramasında dissemine metastatik lezyonları olan bir hastayı sunuyoruz. Her iki akciğerde artmış ^18^F-FDG tutulumu gösteren multipl nodüler lezyonlar vardı. Özefagus torakal segmentte, intraluminal alana taşan ılımlı düzeyde ^18^F-FDG tutulumu gösteren yumuşak doku lezyonu vardı. Asimetrik mide duvar kalınlaşmasında ve safra kesesi duvarında lümeni dolduran yumuşak doku lezyonlarında artmış metabolik aktivite görüldü. Karaciğerde multipl hipodens/hipermetabolik lezyonlar görüldü. Hemen tüm kolon segmentlerinde multipl hipermetabolik polipoid/yumuşak doku lezyonları görüldü. Tüm abdominal kadranlarda multipl hipermetabolik peritoneal implantlar saptandı. Sağ sürrenal bez yumuşak doku lezyonunda artmış ^18^F-FDG tutulumu tespit edildi. Rektumun arka duvarında hipermetabolik yumuşak doku lezyonu vardı. Torakal ve lomber vertebralarda, sol skapulada, sol iliak kemikte, sakrumda ve sol femur boynunda hipermetabolik litik lezyonlar görüldü. Atenüasyon düzeltmesi yapılan ve yapılmayan görüntülerde, ^18^F-FDG tutulumu olan deri lezyonu lehine bulgu yoktu.

## Figures and Tables

**Figure 1:**
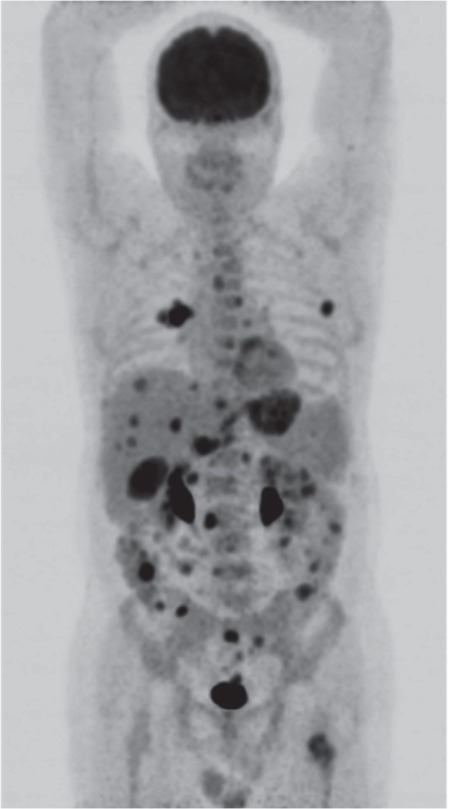
^18^F-FDG maximum intensity projection image. Colonoscopy examination of a 51-year-old man, whose only complaint was severe rectal bleeding, revealed multiple, large necrotic polypoid lesions in all colonic segments. Excisional biopsy has been performed from the sigmoid region. Histopathologic findings and immunohistochemistry analyses including S-100, HMB45 and vimentin were positive and all these findings strongly suggested colonic malignant melanoma. Ophthalmologic, dermatologic and ear-nose-throat examinations were negative for primary melanoma or any melanocytic lesion, thus the case was diagnosed as a colonic malignant melanoma. Melanomas within the gastrointestinal (GI) tract are usually metastatic in origin (1). However, some colonic melanomas are true primary tumors. The probable genesis of such tumors involves a concept of “ectodermal differentiation” - that ectodermal cells are capable of differentiation into multiple cell lines and may variably migrate into the colon during embryologic stages to develop into melanocytes (2). Primitive stem cells localized within the GI tract wall may also give rise to heterotopic melanocytes in the colon (3).

**Figure 2:**
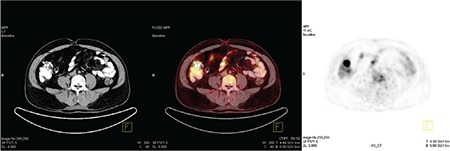
Markedly increased ^18^F-FDG uptake is seen in soft tissue lesions of the colonic hepatic flexure, which was the largest lesion in this patient.

**Figure 3:**
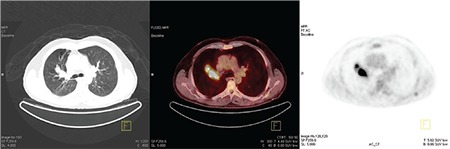
Increased ^18^F-FDG uptake is detected in the lobulated contoured mass lesion at the right upper lung lobe anterior segment.

**Figure 4:**
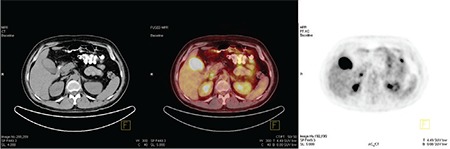
Hyper-metabolic soft tissue lesion located in the gallbladder wall and the entire lumen is viewed.

**Figure 5:**
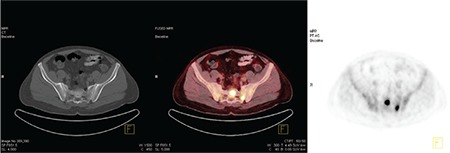
Hyper-metabolic, lytic lesions are visualized at the sacrum.
